# Erratum for Firrman et al., “An *in vitro* model of the small intestinal microbiota provides key insights into interindividual variability in structure and function”

**DOI:** 10.1128/msystems.00999-26

**Published:** 2026-08-05

**Authors:** Jenni Firrman, LinShu Liu, Karley Mahalak, Johanna M. S. Lemons, Adrienne Narrowe, Elliot S. Friedman, Gary D. Wu, Tom Van de Wiele

## ERRATUM

Volume 11, no. 3, e01373-25, 2026, https://doi.org/10.1128/msystems.01373-25. Byline: Tom Van de Wiele’s name should appear as shown in this Erratum.

